# Potential Adsorption Affinity of Estrogens on LDPE and PET Microplastics Exposed to Wastewater Treatment Plant Effluents

**DOI:** 10.3390/ijerph192316027

**Published:** 2022-11-30

**Authors:** Noura Al-Jandal, Abdulaziz AlKhubaizi, Talat Saeed, Mariam Hajeyah

**Affiliations:** Environmental Pollution and Climate Program, Environment and Life Sciences Research Centre, Kuwait Institute for Scientific Research, P.O. Box 24885, Safat 13109, Kuwait

**Keywords:** microplastics, adsorption, influent, effluent, wastewater treatment plant, estrogens

## Abstract

Microplastics (MPs) are among the most common pollutants in the environment. Because of their small size, availability, and similarity to natural foods, they are commonly ingested by marine organisms. They can cause health problems in living organisms due to their bioaccumulation potential. It is, therefore, unknown whether endocrine-disrupting chemicals (EDCs), in particular estrogens, are capable of adhering to the diverse types of MPs found in water. Two MP polymers (low-density polyethylene (LDPE) and polyethene terephthalate (PET)) that could pose a threat to fish were tested for estrogen adsorption. The adsorption capacity of MP pellets was studied for 30 days in the effluent and influent of a wastewater treatment-plant. A laboratory simulation was conducted to validate the field and laboratory findings. We found that the concentrations of five types of estrogen ((diethylstilbestrol (DES), estrone (E1), 17β-estradiol (E2), estriol (E3), 17α-ethinyl estradiol (EE2)) were higher in the influent than the effluent streams. LDPE and PET MPs exposed to influent water in the laboratory had higher estrogen levels than wastewater treatment plants (WWTP) pellets. The PET pellets showed the highest adsorption affinity to EE2, while the LDPE pellets showed the highest affinity to E2. As a result, this study provided baseline data to investigate the estrogen adsorption capacity in MPs.

## 1. Introduction

The global plastic production rate is estimated at 300 million metric tons annually.

Packaging waste, which is distributed widely throughout industry, is considered the primary source of mismanaged plastic waste, as it makes up the majority of plastic produced [1,2]. Insufficient disposal and recycling management results in plastics becoming waste because of the massive level of plastic production [3,4,5]. In the environment, plastic particles are subjected to various environmental factors such as physical abrasion, photochemical weathering, and microbial degradation, ultimately decomposing into smaller parts and fragments called microplastics (MPs), which have become distributed in oceans around the world in recent years [6,7,8,9]. MPs can enter the marine environment directly as granules, primarily employed in manufacturing plastic products (plastic resins), or indirectly through the breakdown of large plastic pieces [10]. One of the primary sources of MPs entering the environment is wastewater treatment plants (WWTPs) [11]. MPs might be considered to be a “cocktail of pollutants,” either included during plastic production or absorbed from waterborne contaminants [12,13]. MPs’ hydrophobic properties and relatively large surface area to volume ratio make them essential surfaces for a wide range of compounds, such as persistent organic pollutants [14,15,16]. Hydrophobic organic contaminants can be incorporated into marine plastic pellets in two different ways: either through adsorption from seawater onto the pellet’s surface due to its hydrophobic nature (low polarity) [17], or through synthetic plastic additives and related chemicals contained in resins, which can potentially harm wildlife [14]. Numerous studies have shown that several chemical compounds can absorb onto the surface of MPs, hence increasing their toxicity to the ecosystem [18]. Considerable research has attempted to identify and quantify MPs from wastewater influent and effluent. The concentration of MPs in the influent of a WWTP ranged between 15 and 640 particles L^−1^, while the concentration in effluents was significantly lower yet varied across four orders of magnitude [19,20,21,22,23,24,25,26,27,28,29,30].

MPs have caused severe environmental concerns since they can absorb toxins and act as a vector of harmful pollutants [31]. Due to their microscopic size, these particles may be swallowed by marine organisms, posing a health risk [32]. As marine fish passively drink seawater to maintain homeostasis, MPs can be absorbed through the ingestion of very small particles of water [33]. Microplastics are ingested by fish either directly or indirectly via trophic transfer, since fish are prone to mistaking MP particles for food [34]. It has been reported that MPs can be found in the stomach, liver, gills, and edible parts of fish that humans consume [35,36,37,38,39].

Furthermore, the feeding approach significantly impacts on MPs’ ingestion rate, particle size, and type [34]. MP consumption can cause oxidative stress, cell damage, tissue inflammation, longer gastrointestinal residence durations, and leaching of chemical additives and adsorbed pollutants [40,41]. The sorption potential of polymers varies; for example, polyethylene pellets have a higher affinity for polychlorinated biphenyls than polypropylene [42]. Even though there have been many studies on MPs’ sorption and the possible effects on the environment [43,44], there is still a lot to learn about MPs and hydrophobic organic compounds, particularly endocrine-disrupting chemicals (EDCs); since aquatic organisms may consume MPs, this is crucial to determining how they affect the marine environment.

Polystyrene (PS), low and high-density polyethylene (PE), polyvinyl chloride (PVC), polypropylene (PP), nylon 66 (PA66), and polyethylene terephthalate (PET) are the most utilized and identified synthetic plastics in the aquatic environment [42,45,46]. According to documented studies, the most abundant plastic polymers in the Arabian Gulf are low-density polyethylene (LDPE), PP, and PE [47,48,49,50]. Synthetic polymers such as PE and PP are light enough to float and spread in seawater [10]. On the other hand, denser polymers such as PVC, PET, and PA66 tend to settle at lower sea levels and near their source point in the environment, but they can still be moved by the currents [51,52]. This is important when considering fish feeding-behavior.

There has been previous research on the adsorption potential of various compounds on plastics. These investigations have mainly been conducted in the laboratory [53]. Therefore, further research is needed, especially in the Arabian Gulf region, where WWTPs receive substantial amounts of plastic components that can reach the marine environment and harm marine life. In addition, MPs might transfer toxic chemicals to marine organisms, particularly fish, increasing their toxic effect. As a result, the current study was developed to examine the adsorption capability of five estrogens ((diethylstilbestrol (DES), estrone (E1), 17β-estradiol (E2), estriol (E3), and 17α-ethinyl estradiol (EE2)). Estrogens have been reported to pose estrogenic effects on the endocrine system of a living organism, such as their growth and reproduction [54,55,56,57,58]. This study will investigate the adsorption of estrogens on LDPE and PET MPs when exposed to influent (inflow stream) and effluent (outflow stream) in Kabd WWTPs. Previously, the estrogen levels in Kabd WWTP influent and effluent streams were examined. The results showed that the average influent levels were 30.2 ng/L, and the average effluent levels were 26.4 ng/L [59]. Another recent study investigated the same WWTP and recorded an estrogen concentration in the influent streams ranging from 0.0 to 474 ng/L, while in the effluent streams, this concentration was between 0.0 and 233 ng/L. In addition, the total estrogen average removal rate was 13% [60,61,62]. Accordingly, Kuwait’s coastal areas have been contaminated with phthalates, alkylphenols, and estrogens [59] when receiving these effluents.

There is insufficient research on toxic chemicals that adhere to microplastics. Researchers should investigate how MPs can acquire chemicals from ambient surroundings and how they might pose a threat. The results of this study will contribute to science and understanding by providing evidence that MPs may act as vectors for EDCs in the marine environment.

## 2. Materials and Methods

### 2.1. Microplastics

This study used MP resin pellets (virgin) with relatively uniform dimensions instead of other shapes such as fragments, fibers, or films. This was essential to avoid differences in the surface area for the chemical exposure experiment. The MPs selected were LDPE and PET due to their widespread use in many different parts of the world today. These pellets (3 mm) (Grand Polymer Co. Ltd., Tokyo, Japan) were purchased from a local distributor at Kuwait City (Kuwait Plastics & Mats; National Industries Company; and Plastic Industries Company).

### 2.2. Microplastic Exposure in Wastewater Treatment Plant

In order for MPs to be prepared for the experiment in Kabd WWTPs, stainless steel mesh sacks were used to hold them during the investigation. Mesh sacks containing MPs were transferred to the plant and securely placed in the inflow and outflow of the plant to receive influent and effluent streams for 30 days. Following 30 days, the MPs were reclaimed and stored in glass vials at 30 °C in the laboratory until further investigation.

### 2.3. Experimental Validation for Quality Control

Simultaneously, a simulation experiment was conducted in the laboratory to determine whether there was a substantial difference between the laboratory and WWTP exposure. Water samples from the influent and the effluent of Kabd WWTP were collected and transferred into the laboratory. Flasks containing 250 mL wastewater samples and 6 g of MPs were kept on a laboratory shaker for 30 days. The wastewater samples collected within the flasks were replaced once every seven days. Virgin MPs (control) were separated and evaluated separately.

### 2.4. Analytical Procedures

MPs pellets were air-dried for three days before chemical extraction. Before use, all glassware was cleaned multiple times with methanol, dichloromethane, and distilled hexane to remove organic contamination. Dried MPs were weighed before being extracted with methanol using ultrasonication. The evaporated extract was transferred into thick-walled V-shaped vials and evaporated to dryness under nitrogen purge. BSTFA/TMCS silylation reagent with pyridine was used to derivatize estrogens and alkylphenols. The vials were heated on a hotplate for 1 h at 70 °C and then cooled to ambient temperature before evaporating to dryness under nitrogen. The residue was dissolved in one milliliter of iso-octane and transferred to an autosampler vial. All samples were processed in duplicate to ensure reproducibility. Gas chromatography–mass spectrometry (GC-MS) was used to examine the samples in the selected ion monitoring mode [59].

### 2.5. Statistical Analysis of Data

Calculations and comparisons were executed using descriptive statistics for the estrogens that were the focus of the study. These statistics comprised the mean values that were measured for each exposure condition. On the plastic pellets, the measured amounts of estrogen were recorded in terms of ng g^−1^. The Shapiro–Wilk test was performed on the data to evaluate whether they were normally distributed, and the results showed that all of the data were normal. We used paired *t*-tests to analyze the differences in estrogen levels between MPs exposed to influent and effluent streams. To carry out all of the necessary statistical computations, Microsoft Excel (Office 365) and IBM SPSS were utilized (v.25).

## 3. Results

### 3.1. Estrogen Concentrations Detected on LDPE and PET Microplastic Pellets

**Figure 1:**
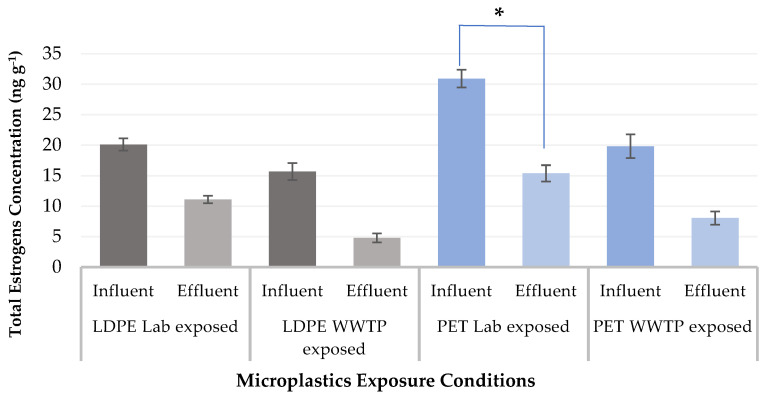
Total estrogen concentrations (ng g^−1^) in WWTP and laboratory exposure settings. Significant differences were detected with a paired *t*-test (* *p* < 0.05). Gray-coloured bars represent LDPE MPs and blue-coloured bars represent PET MPs.

**Figure 2:**
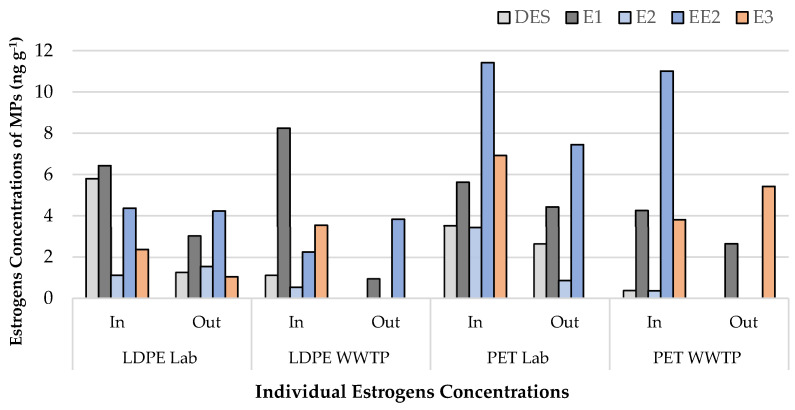
Individual estrogen concentrations in ng g^−1^ in WWTP and laboratory conditions. Lab: laboratory exposed; WWTP: wastewater treatment plant; In: influent; Out: effluent; DES: diethylstilbestrol; E1: estrone; E2: 17β-estradiol; E3: estriol; EE2: 17α-ethinyl estradiol.

After 30 days of exposure, the two types of polymers, LDPE and PET, were tested for the presence of estrogen. The results show that the influent’s total estrogens content (DES, E1, E2, E3, EE2) was approximately 30–55% greater than the levels measured in the effluent-exposed pellets (Figure 1). The individual estrogen concentrations measured under various exposure circumstances are shown in Figure 2.

Based on the results, regarding the influent exposure in the laboratory-exposed settings, PET MPs demonstrated the highest total estrogen level of 30.91 ng g^−1^, followed by the LDPE MPs at 20.09 ng g^−1^. Regarding the effluent exposure in the WWTP, LDPE MPs had the lowest estrogen concentration (4.79 ng g^−1^), followed by the second lowest concentration being detected in PET MPs (8.06 ng g^−1^) (Table 1), indicating the satisfactory removal efficiency of estrogen in the plant streams.

The results of individual estrogens showed that EE2 concentrations were highest in influent-exposed PET MPs under laboratory conditions (11.42 ng g^−1^) and 11.01 ng g^−1^ in WWTP exposure. On the other hand, the highest level of E1 was found in LDPE MPs exposed to influent in both exposure settings (8.24 ng g^−1^ in WWTP and 6.43 ng g^−1^ in the laboratory). Interestingly, E2, E3, and DES concentrations were found to be relatively lower under all exposure circumstances, probably due to their limited adsorption on LDPE and PET pellets or the high removal effectiveness of WWTP (Table 1). Virgin pellets were also analyzed, and the results showed non-detectable levels of estrogens for all the individual estrogens (Table 2).

### 3.2. Elimination Efficiency of Estrogens under Influent and Effluent Treatment Conditions

We used a paired *t*-test to compare the obtained data between the influent and effluent to determine the efficacy of WWTP estrogen removal. Our findings indicate a considerable elimination of estrogens between the influent and effluent measured in PET MPs during laboratory exposure (*p* = 0.04). The other sets were decreased non-significantly. Lower estrogens levels were found in the effluent compared to influent, indicated indirectly by the measured levels of estrogens in both exposed MP types.

### 3.3. Analysis of Virgin MPs

As a precautionary measure, virgin MPs were tested in duplicate to ensure they were free of estrogen and that the estrogen detected in the laboratory and WWTP was derived from the influent and effluent streams. Upon analysis of the virgin pellets, it was revealed that there were no detectable estrogen levels for each of the individual estrogens in the pellets. The estrogen content of virgin pellets is shown in Table 2.

## 4. Discussion

In this study, it was demonstrated that LDPE and PET MPs have the ability to adsorb EDCs, which are contaminants of concern. Among the different classes of EDCs, natural and synthetic estrogens, such as E1, E2, E3, and EE2, are known to be potent [63]. WWTPs are a primary source of steroid hormones released into the marine environment. However, WWTPs cannot completely remove estrogen during the treatment process. [64,65]. Hence, there is a limited source of information available about their possible toxicity route to ecosystems, efficient treatment in WWTPs, and methods of adsorption of other pollutants on the MP surface. In this regard, the present study was primarily focused on investigating the adsorption potential of estrogens on two distinct types of MPs pellets that could serve as contaminants’ vectors.

Based on our findings, the total estrogen levels were 30.5% higher in the influent stream than in the effluent of LDPE MPs exposed to WWTP and 44.04% higher in the influent stream than in the effluent of PET MPs exposed to WWTP. This could be due to the lower levels measured in both streams, indicating that estrogen removal is occurring. In a previous study, 13% of estrogens were removed from Kabd WWTP, which could support this assertion [60,61]. Another study reported higher removal rates of E1, E2, and EE2 in WWTP of 61.76–87.25%, 50.98–82.63%, and 66.3–90.25%, respectively [66]. This explains the higher levels detected in both MPs in the influent compared to the effluent water. The octanol–water partition coefficient for steroid hormones is logK_OW_ > 2, indicating that they can be adsorbed on organic matrices including the particles in sewage treatment plant effluents, such as MPs [67]. The results generally showed that estrogens were partially removed and MPs can act as estrogen transporters; if consumed, they pose a health risks to living organisms.

Many factors can affect the sorption and desorption mechanism kinetics, such as matrix type, degradation, structure, biofilm growth, surface properties, pH, temperature, pollutant solubility, crystallinity, polarity, and chemical bonding [44,68,69,70,71,72,73,74]. A study found that the sorption capacity of PE was the highest due to its larger surface area and more significant gaps between its polymer chains, which lead to diffusion. PET, however, has the highest sorption rate because of its small surface area and glassy polymeric structure that hinders diffusion into the material [75,76,77]. Therefore, the MPs’ nature has a significant role in altering the adsorption affinity.

Although the experiments were conducted carefully and systematically, this study had flaws in that only one WWTP was considered, and a small sample size (two MP polymers) was used. Despite that, this study has several strengths, including the data which provided reliable and robust evidence to help us understand MPs as an estrogen vector and how different polymers can adsorb contaminants. According to the obtained results, some estrogens have a higher affinity for specific polymers, which opens the possibility of investigating pollutant competition. Moreover, this study is the first to investigate estrogen adsorption on MPs in the Arabian Gulf region. Furthermore, estrogens were effectively removed from the effluents, which indicates that the WWTP is operating at a satisfactory level of efficiency. The study also provides a baseline for how estrogen binds to MPs, meaning that we can study the possible effects of MP uptake by marine organisms.

## 5. Conclusions

The toxic contaminant concentrations in the Arabian Gulf region are high. WWTPs receive a considerable number of plastics released straight into the marine environment, besides being the primary source of toxic chemicals. Studies have claimed that MPs serve as vectors for toxic chemicals transported within the environment. However, more studies are needed regarding the adsorption efficiency of EDCs, particularly estrogens, on other types of MPs present in water. We investigated whether estrogens could adsorb on LDPE and PET MPs, that could be a potential threat to marine organisms. Analytical techniques are used to quantify the adhesion of estrogens to microplastic surfaces.

There currently needs to be more studies on EDCs’ adsorption and desorption mechanisms onto and from MPs. Therefore, a better understanding of EDCs’ competitive behavior and selectivity towards MP polymers is required. Research that reveals the bioavailability and uptake of EDCs by MPs could be beneficial in highlighting their ecotoxicological consequences. In conclusion, the five analyzed steroidal hormones, known to occur in marine environments at concentrations of nanograms per liter and that affect marine organisms, are capable of being transported through MPs. Our study suggests that MPs can transfer some potent EDCs within the marine environment. Thus, it will be imperative to recognize MPs as emerging vehicles for contaminant transmission. Hence, future research should explore the sorption affinity of chemicals to MPs under several environmental conditions and investigate their toxicity on a physiological and molecular level in marine organisms.

## Figures and Tables

**Table 1:** Estrogens (ng g^−1^) on the pellets (ng g^−1^) (level of significance calculated using paired *t*-test between influent (in) and effluent (out); * *p* < 0.05 with two-tailed relevance).

Estrogens	LDPE Lab In	LDPE Lab Out	*p*- Value	LDPE WWTP In	LDPE WWTP Out	*p*- Value	PET Lab In	PET Lab Out	*p*- Value	PET WWTP In	PET WWTP Out	*p*- Value
DES	5.80	1.27	0.13	1.13	0.00	0.22	3.52	2.65	0.04 *	0.39	0.00	0.37
E1	6.43	3.04		8.24	0.96		5.62	4.43		4.25	2.64	
E2	1.12	1.54		0.54	0.00		3.43	0.86		0.36	0.00	
EE2	4.37	4.23		2.24	3.83		11.42	7.45		11.01	0.00	
E3	2.37	1.04		3.54	0.00		6.92	0.00		3.81	5.42	
Σ Estrogens	20.09	11.13		15.68	4.79		30.91	15.38		19.82	8.06	

Lab: Laboratory setting; WWTP: wastewater treatment plant; In: influent (inflow); Out: effluent (outflow); DES: diethylstilbestrol; E1: estrone; E2: 17β-estradiol; E3 estriol; EE2: 17α-ethinyl estradiol.

**Table 2:** Estrogens in LDPE and PET virgin pellets.

Microplastic Virgin Pellets	DES	E1	E2	EE2	E3
PET Sample 1	ND	ND	ND	ND	ND
PET Sample 2	ND	ND	ND	ND	ND
LDPE Sample 1	ND	ND	ND	ND	ND
LDPE Sample 2	ND	ND	ND	ND	ND

ND: non-detectable.

## Data Availability

All the data generated or analyzed during this study are included in this article.
